# Evaluation of Packaging Materials in Freeze-Drying: Use of Polymer Caps and Nested Vials and Their Impact on Process and Product Attributes

**DOI:** 10.1208/s12249-021-01953-8

**Published:** 2021-02-23

**Authors:** Tim Wenzel, Henning Gieseler

**Affiliations:** 1 Department of Pharmaceutics, Freeze Drying Focus Group (FDFG), Friedrich-Alexander University (FAU) Erlangen-Nuremberg, Cauerstrasse 4, 91058 Erlangen, Germany; 2 GILYOS GmbH, Friedrich-Bergius-Ring 15, 97076 Würzburg, Germany

**Keywords:** freeze-drying, packaging material, nested vials, polymer cap

## Abstract

Current trends in the pharmaceutical industry led to a demand for more flexible manufacturing processes with smaller batch sizes. Prepackaged nested vials that can be processed as a unit were introduced into the market to fulfill this need. However, vial nests provide a different thermal environment for the vials compared to a hexagonal packaging array and could therefore influence product temperature profiles, primary drying times, and product quality attributes. Polymer caps with the possibility of vial closure inside the freeze-drying chamber were developed to remove the risks and need of a crimping process. A general concern with the use of such caps is the possibility of an increase in resistance to water vapor flow out of the vial. This case study investigated the effect of the LyoSeal^®^ and PLASCAP^®^ polymer caps and EZ-fill^®^ nests on the freeze-drying process. Amorphous and partially crystalline model formulations were freeze-dried. Process data and product quality attributes were compared for regularly stoppered vials and vials with polymer caps as well as vials in a hexagonal packaging array and nested vials. The results indicated no increased resistance or impeded water vapor flow by the polymer caps. Differences in the macro- and microscopic appearances of products and a trend towards lower product temperatures were observed for the investigated nest type compared to a regular hexagonal packaging array. Consequently, the polymer caps could be used as an alternative to regular stoppers without affecting freeze-drying process data or product quality attributes, while the different thermal environment of nested vials should be considered.

## INTRODUCTION

Freeze-drying is an integral manufacturing technique for the preparation and stabilization of parenteral drugs. Approximately 50% of the approved biopharmaceutical drugs are processed by freeze-drying according to the US Food and Drug Association (FDA) and European Medicines Agency (EMA) (1). After vial filling, stopper placement, and loading of the freeze-dryer, the freeze-drying process itself is performed in three steps. First, the product solution is completely solidified during the freezing step by reducing the shelf temperature (T_s_) at atmospheric pressure. Next, the chamber pressure is reduced to facilitate ice sublimation during primary drying. T_s_ is typically increased during this step to provide the energy required for sublimation while maintaining the product temperature below its critical formulation temperature to avoid cosmetic defects and quality issues. Lastly, unfrozen water that is immobilized in the amorphous product matrix or adsorbed to the product surface is removed by diffusion and desorption during secondary drying by a further increase in T_s_ (2, 3). After the freeze-drying process, the vials are stoppered under vacuum within the freeze-drying chamber before unloading and capping them with an aluminum crimp.

The most common packaging system for parenteral drugs is a glass vial with a rubber stopper and an aluminum crimp. An adequate combination of vial, stopper, and crimp as well as proper control of the capping process itself is critical to ensure container closure integrity (CCI) over the course of the shelf life of the drug product (4, 5). Examples of risks during the crimping process include CCI failure due to inadequate crimping forces (too high or too low), vial breakage during the transport and crimping step, metal particle generation, or cosmetic defects of the crimp (5). Guidelines by the European Commission (6) or the FDA (7) state that the capping process should be performed in an aseptic area or with appropriate assurances to safeguard the product outside of an aseptic area until the cap is crimped.

Because of advances in personalized medicine, there is currently a trend for more flexible manufacturing processes and smaller batch sizes in pharmaceutical freeze-drying (8). To address this, packaging material manufacturers are providing vials prepackaged and ready-to-use in racks or nest systems that can be processed as a unit rather than as singular vials. These systems can help reduce the processing time during filling, loading, and unloading as well as reduce the time necessary to switch between primary packaging materials because the nests are provided in standardized dimensions for different vial sizes (9). With currently available systems, two different design types can be distinguished. The EZ-fill^®^ ready-to-use vials by Ompi are an example of a mold-design, where each vial is placed in a mold (10). This mold-design results in a thermal barrier between the vial bottom and freeze-dryer shelves as well as polymer walls adjacent to the vials that shield them from other vials in the nest and the chamber walls. The other design type holds the vials with a polymer rack that is fixated around the vial necks. Vials are separated from adjacent vials with no physical barriers in between them while still maintaining direct contact between the shelf surface and vial bottom. An example of this design type are the Schott adaptiQ^®^ ready-to-use vials (9). In previous investigations, the effect of the adaptiQ^®^ vial nests on freeze-drying processes has been evaluated. Deutschle and Selch (9) reported an approximately 10% reduction in primary drying time with a 3% mannitol solution at pilot and manufacturing scale with minimally lower residual moisture values in nested vials compared to an array with hexagonal packaging. Daller et al. (8) reported a reduction in the product temperature differential between edge and center vials and that heat transfer is dominated by direct contact between vial and shelf as well radiation from the rack itself for adaptiQ^®^ nests. Although not performed in a nest, a previous study by Kuu et al. (11) investigated the effect of a thermal barrier below the vials on the freeze-drying process in the form of the gap-freezing concept. They have reported higher nucleation temperatures and consequently lower product resistances and faster primary drying times as well as improved macroscopic product appearance for a 10% sucrose solution when processed with a gap between the vial bottom and the shelf. Regarding nested vials with a mold-design type, a study investigating the effect of a freeze-drying cycle where primary and secondary drying was performed in single steps at shelf temperatures of 35°C and 40°C with unspecified chamber pressures on sucrose-based product solutions in hexagonal packaging arrays, an EZ-fill^®^ vial nest and an EZ-fill^®^ vial nest within a tub, was performed and published in an advertorial (12). The authors reported improved macroscopic and microscopic structures for the products processed in the nests because of the thermal barrier provided by them. The detrimental effects of the high shelf temperature during the drying step on hexagonally packed vials with sucrose-based products were to be expected, but the improved appearance of the nested vials highlighted the effect of the nest as a thermal barrier. A similar concept to the mold-design nests can be found in syringes freeze-dried in a custom designed aluminum block or freeze-drying of the VirTis 96-well freeze-drying system for polymerase chain reaction plates: investigations for both systems showed reduced heat transfer compared to freeze-drying in regular vials and the importance of gas conduction, contact conduction, and radiation on overall heat transfer; while radiation was typically reduced by the aluminum block, the containers were placed in compared to hexagonally placed vials (13).

In recent years, manufacturers also introduced several alternatives to the standard combination of stopper and aluminum crimp to the market to address issues with the vial capping progress. The LyoSeal^®^ (LS) by West, RayDyLyo^®^ cap by ARaymondlife, and the PLASCAP^®^ (PC) by Daikyo are examples of this (14–16). These polymer caps utilize the stoppering mechanism of the freeze-dryer to instantly seal the vials inside the freeze-drying chamber. Consequently, any risks related to the crimping process and the need of crimping equipment are eliminated. The LS is placed over a regular rubber stopper and intended to be used in a regular hexagonal packaging array. For reconstitution, the cap features a button at the top that can be removed similar to regular Flip-Off^®^ crimps that exposes the stopper underneath. The PC caps function similarly but are provided with an integrated stopper (16). They are commercially available for liquid fill only. Their assessment and feasibility for lyophilized drugs is ongoing. It is reasonable to assume that the caps themselves might increase the resistance to water vapor flow from vials during the freeze-drying process considering the resistance a stopper imposes depends on the size of the stopper opening (4). IMA Life investigated this effect for the RayDyLyo^®^ caps with vials filled with pure water and pressure setpoints in between 38 and 113 mTorr and published the results in a white paper (15). They found significantly higher product temperatures during primary drying (up to 3.5°C for center vials) and three to four times lower mass flow rates for vials with the caps compared to regularly stoppered vials. The problem of increased resistance to water vapor flow has also been explored with protective bags as a containment solution for highly potent substances: an increased resistance to water vapor flow depending on the permeability of the material has been reported for freeze-drying cycles of vials in bags leading to longer primary drying times as well as increased pressure and product temperatures within the bags (17, 18).

This case study evaluated the influence of LS and PC caps and EZ-fill^®^ nests on freeze-drying process data as well as product quality attributes. The effect of the caps and nests on product temperature during primary drying as well as macro- and microscopic product structure, residual moisture, and crystallinity was analyzed. If the caps were to increase the resistance to water vapor transport during primary drying, the effect would be detectable by product temperature increases or more pronounced collapse and higher residual moisture contents, similar to the previously reported results for other caps or containment systems (15, 17, 18). An amorphous and partially crystalline model system was chosen and processed with drying conditions ranging from conservative to aggressive. The investigated amorphous system was a temperature sensitive formulation that could indicate higher local product temperatures by more pronounced viscous flow or collapse. The partially crystalline system was robust enough to be processed aggressively and evaluate the performance at high mass flow rates. Process data and product quality attributes were analyzed and compared between regularly stoppered and vials with polymer caps as well as vials in a hexagonal array and nested vials. It is important to note that the investigated packaging materials are suited to specific needs and implementation or evaluation should be decided based on these. The purpose of this study was to show practitioners in pharmaceutical freeze-drying what they could expect when confronted with them.

## MATERIALS AND METHODS

### Materials

**Fig. 1:**
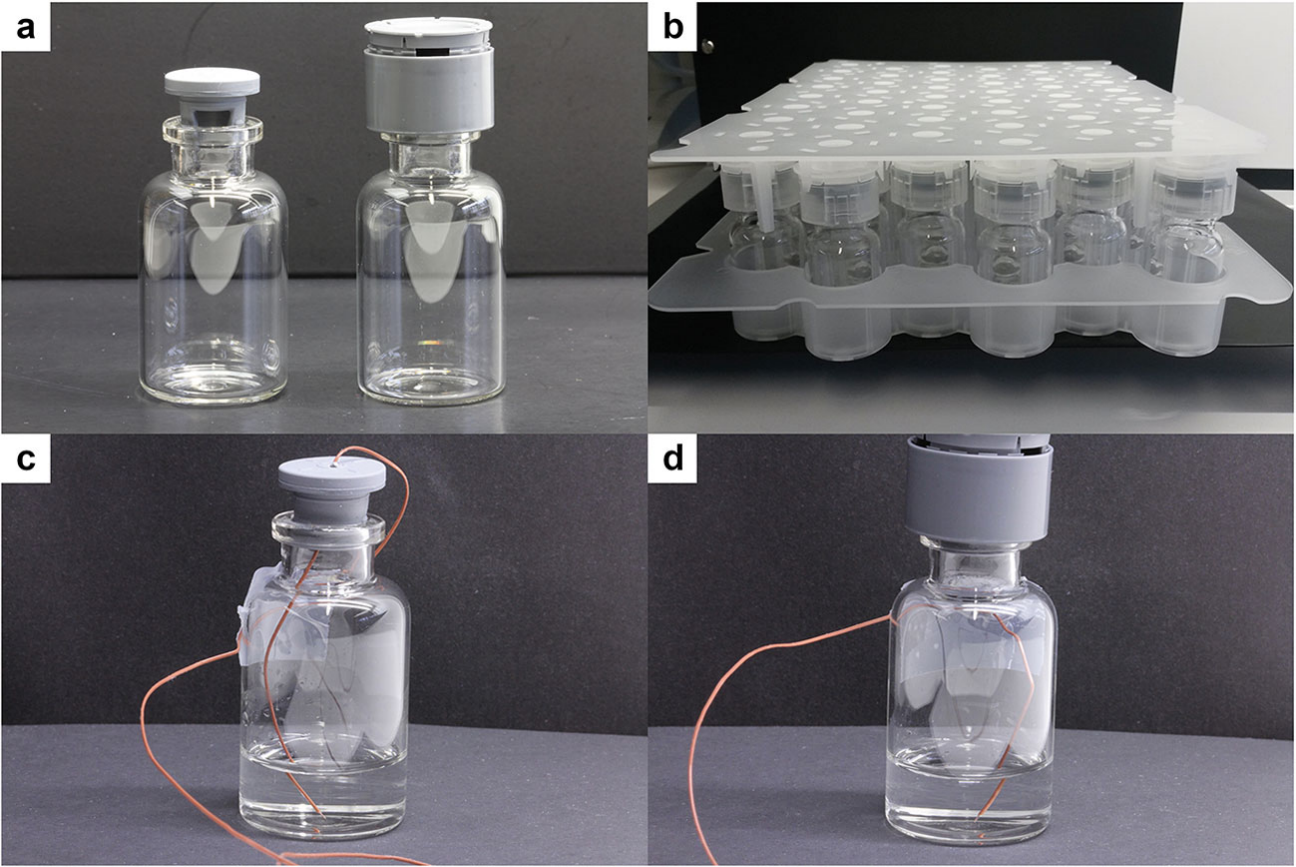
Overview of packaging material used. Regularly stoppered vial and vial with LS cap (**a**), EZ-fill® nest with PC caps (**b**), regularly stoppered thermocouple vial (**c**), and LS thermocouple vial (**d**)

Tubing vials with a 20 mL nominal fill volume by MGlas (Münnerstadt, Germany) were used in the LS experiments. 10 mL and 20 mL tubing vials by Ompi (Piombino Dese, Italy) were used in the PC experiments. 20 mm Westar^®^ RS stoppers (West Pharmaceutical Services, Exton, PA) were used in conjunction with the LS as recommended by the manufacturer and for the regularly stoppered vials in the LS experiments. Vials in the PC experiments were stoppered with 20 mm Daikyo RUV^®^ stoppers supplied by Daikyo Saiko (Tokyo, Japan). All regularly stoppered vials were sealed with 20 mm Flip-Off^®^ seals (West Pharmaceutical Services). The LS and PC polymer caps were provided by West and Daikyo, respectively. The LS caps in the tested version were not commercially available at the time of the experiments. Ompi EZ-fill^®^ nests were used in the experiments with nested vials. Example images of the LS and an EZ-fill^®^ nest with 10 mL vials and PC caps are shown in Fig. 1a and b, respectively.

S-Adenosyl-L-methionine disulfate tosylate (SAM) was obtained from Shaanxi Sciphar Natural Products (Xi’an, China). D-Mannitol was purchased from Sigma-Aldrich (Munich, Germany). Water for injection (WFI) was obtained from B Braun (Melsungen, Germany). Milipak^®^-20 filters with a 0.22 μm pore size were bought from Merck (Darmstadt, Germany). Calibrated 36 AWG thin-wire type T thermocouples (TCs) were purchased from OMEGA Engineering (Deckenpfronn, Germany) for temperature monitoring.

### Methods

#### Compounding and Freeze-Drying

Two formulations were investigated throughout the course of this study: an amorphous system with 100 mg/mL SAM (F1) and a partially crystalline system with 30 mg/mL SAM and 70 mg/mL mannitol (F2). F1 represented an amorphous system that is sensitive to product temperature deviations and mainly functioned as an indicator for differences in macro- and microscopic structure and related product quality attributes. Its low collapse temperature of −36.4°C was essential for the purpose of this study because of its susceptibility to macro- and microscopical defects and the necessity of low chamber pressures during primary drying (19). F1 was a worst-case model system for the polymer cap evaluation because of the increased likelihood of impeded water vapor flow at lower chamber pressures (20). F2 was chosen as a robust model system and mainly served as an indicator for differences in crystallinity and process data. The robustness of the microscopic pore morphology of the partially crystalline F2 enabled comparisons of product temperature data up to aggressive drying conditions without introducing variability due to collapse or shrinkage. The compounds were dissolved in WFI and the solutions sterile-filtered with a Milipak^®^-20 filter with a nominal pore size of 0.22 μm. The fill volume was adjusted to the vial size to obtain a fill depth of 1 cm (6.0 mL for 20 mL vials, 3.8 mL for 10 mL vials).

The solutions were freeze-dried in a LyoStar™ freeze-dryer (SP Scientific, Gardiner, NY) with one shelf latched. An overview of the performed freeze-drying cycles is shown in Table I. Two shelves were loaded in each experiment. LS or PC vials were placed on the top shelf, while the regularly stoppered vials were placed on the bottom shelf. The hexagonal vial arrays were surrounded by empty vials to provide additional radiation shielding (2, 21). Two EZ-fill^®^ nests were placed in the back and front center of each shelf for the cycles with the nested configuration. The number of filled vials for each cycle was adjusted to avoid a loss of chamber pressure control (choked flow conditions) at the relatively low chamber pressures used in the experiments (2). TCs were placed invasively in the center touching the vial bottom to measure the product temperature at the vial bottom (T_b_). For regularly stoppered vials, TCs were introduced through the stopper (Fig. 1c), while modified vials with a 1 mm hole near the vial neck were used for LS and PC vials (Fig. 1d). The hole was taped over so that it would not affect the resistance to water vapor flow out of the vial.

**Table I:** Overview of the Experiments and Primary Drying Parameters

Experiment	Vial size (mL)	Packaging array	Investigated closure system	Shelf temperature setpoint (°C)	Chamber pressure setpoint (mTorr)
L1	20	Hexagonal	LS	-20	28
L2	20	Hexagonal	LS	-15	40
L3	20	Hexagonal	LS	0	40
L4	20	Hexagonal	LS	+25	40
P1	20	Hexagonal	PC	-20	28
P2	20	Hexagonal	PC	+25	40
P3	10	Nested	PC	-20	28
P4	10	Nested	PC	+25	40
P5	10	Nested	PC	-5	100

The shelf temperature (T_s_) was decreased to −45°C with 45-min equilibration steps at +5°C and −5°C and held for 90 min. Afterwards T_s_ was increased to −15°C, and the samples were annealed for 8 h to minimize inter-vial heterogeneity and facilitate complete mannitol crystallization in F2 (22). The freezing step was concluded by decreasing T_s_ to −45°C and holding it for 90 min. Primary drying was performed at the setpoints listed in Table I. The relatively low chamber pressures were necessary because of the low collapse temperature of F1. Chamber pressure and T_s_ setpoints during L1 and P1 were chosen based on previous experiences with F1 (19). The chamber pressure was increased to 40 mTorr in the other cycles to create more defects in F1 and allow for higher mass flow rates by a T_s_ increase without the loss of chamber pressure control due to choked flow. T_s_ during L2, L3, and L4 was successively increased to produce higher mass flow rates as well as provoke more defects in F1. Based on the results of the LS experiments, only the extreme process conditions of −20°C T_s_ and 28 mTorr as well as +25°C T_s_ and 40 mTorr were performed with the PC. The process conditions in P5 were chosen based on the results of the other PC cycles. All T_s_ changes during freezing and primary drying were controlled at 1°C/min. Secondary drying was initiated when the Pirani sensor reached chamber pressure (23). The chamber pressure remained unchanged during secondary drying, and T_s_ was increased to 45°C with 0.1°C/min and held for 6 h.

#### Optical Inspection

**Fig. 2:**
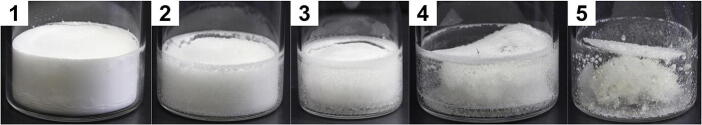
Example images for the defect classification

All products were macroscopically inspected using an Apollo 2 Liquid Viewer (Adelphi Manufacturing, Haywards Heath, UK). Defect classes between 1 and 5 based on the extent of shrinkage and collapse observed were defined with 1 representing an ideal, elegant product and 5 a fully collapsed structure. Example images of each defect class are shown in Fig. 2. A defect class was assigned to each product, and the number of products per defect class was compared for LS or PC and regularly stoppered vials.

#### Scanning Electron Microscopy

The inner pore morphology was assessed by scanning electron microscopy (SEM). Product cakes were carefully extracted from vials with a custom made cutter. The cakes were split in half and fixated on aluminum stubs. Samples were gold sputtered with a Hummer I sputter system (Anatech USA, Union City, CA) at 4 mA for 20 min total. The samples were analyzed with an Amray 1810 scanning electron microscope (Amray Inc., Bedford, MA) using an acceleration voltage of 10 kV. Two vials per defect class were analyzed for each formulation, sealing solution, and cycle.

#### Residual Moisture

Karl Fischer titration was performed with an 831 KF Coulometer. Sample preparation was performed in a glovebox at <1% rH. Product cakes were homogenized, and approximately 100 mg was transferred and sealed in an analysis vial. Water was extracted from the samples by heating in an 832 KF Thermoprep oven system (Deutsche METROHM GmbH & Co. KG, Filderstadt, Germany) while purging with dry nitrogen at 60 mL/min. An oven temperature of 75°C was found to be optimal for analysis since higher temperatures resulted in product degradation. Three vials were analyzed for each formulation with and without LS or PC. One titration was performed per vial.

#### X-Ray Powder Diffraction

Crystallinity was analyzed by X-ray powder diffraction (XRPD) with an X’pert diffractometer (PANalytical B.V., Almelo, Netherlands). The sample chamber was purged with dry nitrogen at 75 mL/min during analysis. Diffractograms were scanned over a range of 0° to 40° 2θ with 0.02° 2θ steps and 1 s/step.

#### Statistical Analysis

The LS or PC vial data was statistically compared to the data of regularly stoppered vials. Differences in T_b_ and residual moisture were statistically analyzed by a Welch’s *t*-test on a 95% confidence level (24). *p*-values below 0.05 were considered statistically significant.

## RESULTS AND DISCUSSION

### General Handling

During manual placement, the operator must be careful not to push in the stoppers when placing the LS over them. This concern was eliminated by the integrated stopper design of the PC. The reader is advised that the caps required a higher stoppering force of the freeze-dryer shelves to close properly compared to regularly stoppered vials.

Invasive instrumentation could be placed by two routes. One possibility would be to remove the button on the top of the caps that is normally removed for reconstitution to expose the stopper underneath and enter the probes through the stopper identically to regularly stoppered vials. For this study, we opted for modifying the vials themselves and drilled a small hole near the vial neck to not alter the characteristics of the caps themselves. Both options required manual interaction. Wireless sensors would be ideal for the use of these caps in clean room environments and automatic loading systems but would also require custom solutions for the antenna in the case of the Tempris^®^ or WTMplus sensors (25, 26).

Another factor that needs to be considered is the size of the caps. As visible in Fig. 1, both caps introduced extra height to the vials. The height difference for an LS vial compared to a regularly stoppered vial was approximately 1 cm. In contrast, the PC with its integrated stopper only added approximately 0.2 cm to the vial height. In practice, these differences could necessitate different shelf separation distances and ultimately lead to smaller possible batch sizes in a freeze-dryer. Additionally, both caps are slightly wider (25 mm) than the vial diameter for 10 mL tubing vials (24 mm). This is no issue in a nested configuration, as intended for the PC, but prevents usage of them in a hexagonal packaging array with vial sizes smaller than 20 mL.

### Freeze-Drying Process Observations

**Fig. 3:**
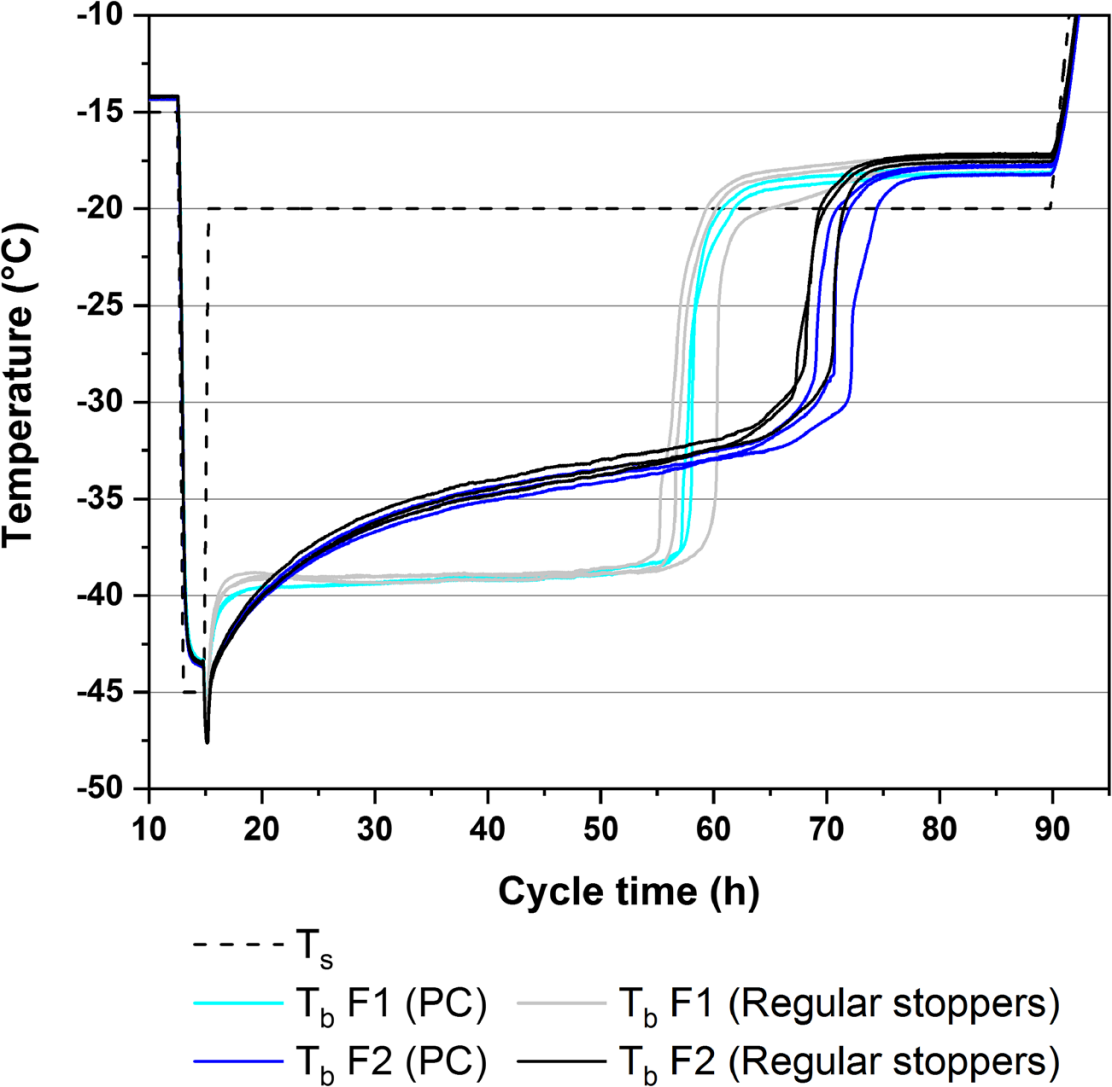
Product temperature data during primary drying for the cycle P1 for PC and regularly stoppered vials

**Fig. 4:**
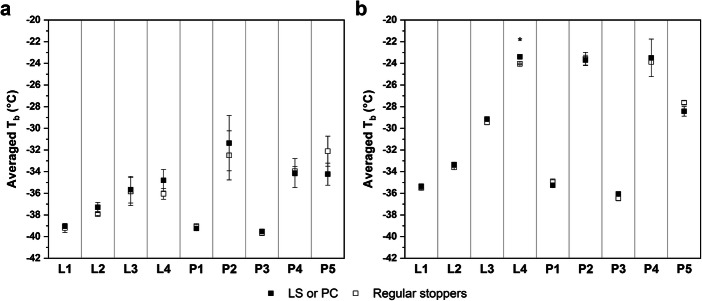
Averaged T_b_ data and standard deviation for F1 (**a**) and F2 (**b**) during primary drying for all experiments. Statistically significant differences between LS or PC and regularly stoppered vials are marked with a star symbol

Primary drying temperature data of the cycle P1 is shown in Fig. 3 as an example. T_b_ for F1 remained constant at approximately −39 °C throughout primary drying. F2 dried at a higher T_b_ which hinted at higher product resistance due to smaller pore sizes of the partially crystalline system. For data comparison between LS or PC and regularly stoppered vials, T_b_ was averaged throughout primary drying from 2 h after the T_s_ setpoint had been reached until before the first TCs of each formulation showed an increase in T_b_. The averaged T_b_ data was compiled in Fig. 4 for all freeze-drying experiments. Concerning the effect of the caps, the only statistically significant difference in T_b_ was found for F2 in the cycle L4. However, the observed difference was so small that it can be considered practically irrelevant.

Only the cycles L1, L2, P1, and P3 controlled T_b_ below the collapse temperature of F1. T_b_ of the other cycles was found either approximately at the collapse temperature or above it. Controlling the temperature in regions where more defects were to be expected was important for evaluating the effects the caps have on the macroscopic structure of the products. A trend towards lower T_b_ values of F1 was observed in cycle L4 compared to P2 despite the same processing conditions, whereas no differences were found for L1 and P1. We hypothesized that this difference could be attributed to differences in the vial systems used in both experiments. Different thermal characteristics could lead to varying collapse behavior that resulted in different drying temperatures of the sensitive amorphous F1, while no measurable effect was observed for the conservative drying conditions or robust partially crystalline F2. Comparison of T_b_ during the PC experiments in the hexagonal (P1 and P2) and nested (P3 and P4) configurations showed a trend towards lower drying temperatures in the nested array with identical drying conditions. This highlighted the additional thermal barrier the nests provided during freeze-drying that needs to be accounted for in a nested setup similar to the gap-freeze-drying concept or custom syringe and 96-well plate holders (11, 13). It should be noted that while nested vials are more accessible to radiative heat transfer due to their separation from neighboring vials, the overall contribution of radiation was reduced by the colder surfaces exposed to the vials. The surface temperature of a rack holding the vials at the neck during primary drying was reported to be approximately 10°C colder than the chamber wall, and Daller et al. (8) reported higher sublimation rates in separated vials without the rack as a radiation shield. This thermal barrier was even more dominant in this study because of the mold-design of the nest type used.

Considering the low chamber pressure of 28 mTorr in the cycles L1, P1, and P3 as well as the aggressive T_s_ setpoint of +25°C and 40 mTorr chamber pressure in the cycles L4, P2, and P4, it is reasonable to assume no measurable influence of the caps on the mass flow rate or T_b_ at higher pressure and lower T_s_ setpoints because of the lower likelihood of impeded water vapor flow at higher pressures or lower sublimation rates (20). Consequently, practitioners will likely not notice a resistance-related effect with other formulations and more moderate process conditions which are more representative for conventional freeze-drying cycles.

### Optical Inspection

All products of F2 were pharmaceutically elegant and classified as defect class 1. This was expected behavior for a partially crystalline system with a high mannitol content. The macroscopic appearance of the amorphous F1 ranged from elegant with minimal shrinkage to total collapse depending on the freeze-drying conditions. The results for F1 were summarized in Table II. The conservative drying conditions used in the cycles L1, P1, and P3 were well suited for F1 and resulted in a good product appearance with only minimal overall shrinkage. A larger number of vials were classified worse in the cycle L2 compared to L1, P1, and P3 despite T_b_ being controlled below the collapse temperature as well. As expected based on the T_b_ values, the higher chamber pressure and T_s_ setpoints led to more macroscopic defects in the cycles L3, L4, P2, P4, and P5.

**Table II:** Number of Vials in Each Defect Class Determined During the Optical Inspection for Formulation 1

Experiment	LS or PC vials					Regularly stoppered vials				
	Defect class					Defect class				
	1	2	3	4	5	1	2	3	4	5
L1	2	34	0	0	0	4	32	0	0	0
L2	3	24	9	0	0	2	16	18	0	0
L3	0	3	14	19	0	0	6	18	12	0
L4	0	0	0	18	0	0	0	2	16	0
P1	0	48	0	0	0	0	48	0	0	0
P2	0	0	1	23	0	0	0	1	23	0
P3	0	48	0	0	0	0	48	0	0	0
P4	0	0	2	22	0	0	0	6	18	0
P5	0	0	3	43	2	0	0	1	36	11

Comparison of the LS and regularly stoppered vials for the cycles L1 to L4 showed varying results. Improved product appearance for the LS vials was found in cycle L2 compared to regularly stoppered vials. The opposite was true for cycle L3, while the results for L1 and L4 were similar. This observation is another indicator that the LS cap did not lead to an increase in resistance to water vapor transport. If the differences were caused by an increase in resistance, the observation would have been in the same direction because an increased resistance would automatically cause lower sublimation rates and as a result higher local product temperatures.

The PC results showed identical macroscopic appearances for the products from the cycles P1 to P3. P4 showed a higher number of vials in worse defect classes for the PC vials compared to the regularly stoppered vials. Because this difference was only observed in the nested configuration in P4 and not during P2 with identical processing conditions, the experiment P5 was added to further investigate the effect of the PC in a nested configuration. During P5, the opposite effect was found with an improved product appearance with the PC caps compared to the regularly stoppered vials. Because the effect was opposite in both experiments and only encountered with the nested configuration, it is likely not caused by an increase in resistance to water vapor transport during primary drying. We hypothesized that the plate the PC caps are arranged in (Fig. 1b) contributes to these differences. For conventionally stoppered vials, the stopper and the top of the vial were exposed to radiative heat from the shelf above; while nested vials with PCs had the plastic plate, the caps were fixated in above them. This plate could absorb and diffuse radiative heat coming from above leading to differences in radiative heat transfer.

### Inner Pore Morphology

**Fig. 5:**
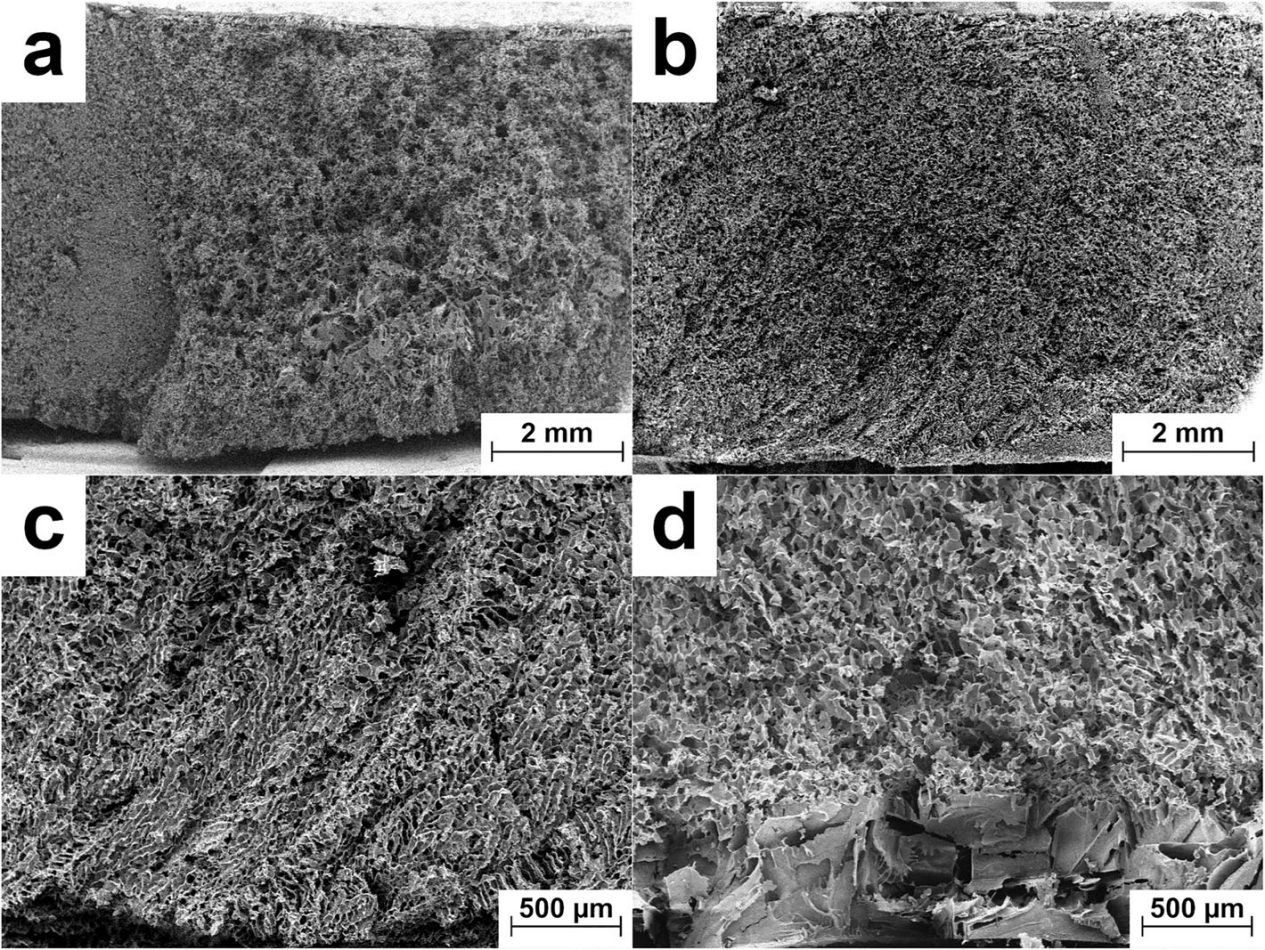
Example images of the inner structure obtained by SEM analysis. Cross section defect class 2 F1 (**a**), cross section defect class 1 F2 (**b**), center bottom section hexagonal array (**c**), and center bottom section nested vial (**d**)

Example images of defect class 2 F1 and defect class 1 F2 samples are shown in Fig. 5a and b, respectively. All defect class 1 and 2 samples for F1 contained a small microcollapse area in the center of the product regardless of which sealing system was used. F2 samples showed a homogeneous pore structure with no signs of viscous flow throughout the entire product as expected from a partially crystalline system with a high mannitol content. The pore sizes for F2 samples were smaller (approximately 50–75 μm) compared to F1 (approximately 75–100 μm) which indicated higher product resistance and explained the higher T_b_ values observed for F2 (27). No differences in the size of the microcollapse area for F1 or the pore sizes for F1 and F2 could be observed between LS or PC and regularly stoppered vials.

A comparison of the pore morphology at the bottom center of the products between the hexagonal and nested configurations is shown in Fig. 5 c and d. All products dried in the nested configuration featured a distinct area with larger pores at the bottom, while pore sizes for the hexagonal configuration were more homogeneous. This showed how a vial nest can influence the freezing behavior of a solution. During freezing, the vial molds resulted in less efficient removal of crystallization heat from the vial and a small fraction of water likely remained unfrozen after nucleation. The unfrozen water remained at the bottom of the product due to its higher density and freezes later when the solution has warmed near the equilibrium freezing point resulting in larger pore sizes in the bottom of the products. This effect was similar to the large-pored region near the bottom of the products observed with controlled ice nucleation at high shelf temperatures which were insufficient for absorbing enough heat for the entire solution to freeze instantaneously (19).

### Residual Moisture and Crystallinity

**Fig. 6:**
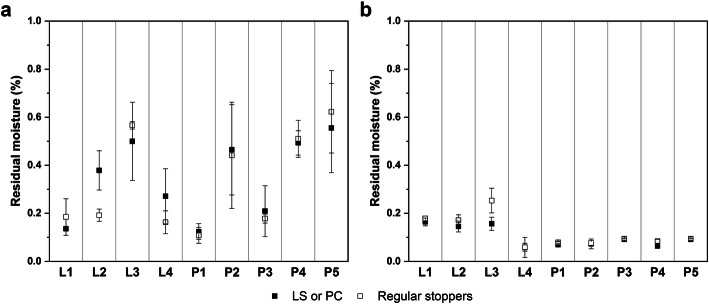
Residual moisture data and standard deviation for F1 (**a**) and F2 (**b**) for all experiments

An overview of the residual moisture content for all products is provided in Fig. 6. All cycles resulted in residual moisture contents well below 1%. Water content in F1 was found below 0.3% in the cycles L1, P1 and P3, and L4. The lower water content in L1, P1, and P3 was expected because of the improved product appearance and the well-described link between collapse and increased residual moisture (28, 29). The low values for the L4 products could have been caused by problems with the homogenization of the samples because the collapsed areas of defect class 4 samples could not be pulverized as well. Generally, the heterogeneity of residual moisture levels was higher for cycles with worse product appearance. None of the measured differences between the capping solutions and regularly stoppered vials were statistically significant.

**Fig. 7:**
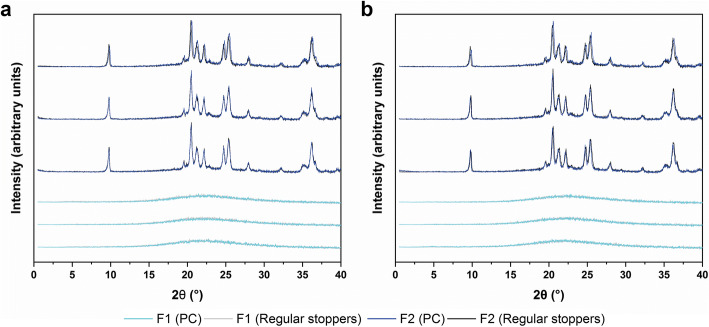
Example diffractograms for products obtained from the cycles P1 (**a**) and P3 (**b**)

Example diffractograms from the XRPD analysis of the cycles P1 and P3 are shown in Fig. 7. The lack of peaks confirmed the purely amorphous nature of F1. The data showed that the mannitol in F2 was successfully crystallized. The peak positions at 10, 19–22, 24–25, and 35–36° 2θ showed the δ polymorph as the main compound (30). No differences in the type of polymorph or the content of each polymorph were observed for LS and PC vials compared to regularly stoppered vials. While an influence of the nests on the freezing behavior and pore structure was confirmed by SEM analysis, comparison of the diffractograms in Fig. 7a and b confirmed that they did not have an influence on the mannitol crystallinity.

## SUMMARY

The LS and PC caps were easy to use and provided instant vial closure within the freeze-drying chamber by means of the regular stoppering mechanism. Vial closure with the LS and PC caps required a higher stoppering pressure than conventional stoppering. The data presented suggested that the caps themselves had no systematic influence on T_b_ during primary drying, residual moisture, the macro- and microscopic product structure, and crystallinity. The EZ-fill^®^ vial nests with a mold-design and no direct contact of vials with the shelves were evaluated for their influence on macro- and microscopic structure and crystallinity. The additional thermal barrier provided by the nests led to an improved macroscopic appearance of an amorphous model system processed with aggressive primary drying conditions. SEM analysis revealed the formation of a distinct large-pored region near the bottom of the products for vials processed in these nests because of their influence on the freezing process. The nests did not influence the mannitol crystallinity in our experiments. It is important to note that the observations in this case study were made with relatively simple model systems. While it is reasonable to assume that problems related to increased resistance to water vapor flow are less likely to occur at higher chamber pressures or lower sublimation rates, the reader is advised that the conclusions drawn for the product quality attributes may not necessarily be valid for other more complex formulations.
